# Snakes and ladders: World development pathways’ synergies and trade-offs through the lens of the Sustainable Development Goals

**DOI:** 10.1016/j.jclepro.2020.122147

**Published:** 2020-09-10

**Authors:** George Philippidis, Lindsay Shutes, Robert M’Barek, Tévécia Ronzon, Andrzej Tabeau, Hans van Meijl

**Affiliations:** a European Commission, Joint Research Centre (JRC), Seville, Spain; b Aragonese Agency for Research and Development (ARAID), Centre for Agro-Food Research and, Technology (CITA), Agrifood Institute of Aragón (IA2), Government of Aragón, Zaragoza, Spain; c Wageningen Economic Research, The Hague, the Netherlands; d Consulting Economist, Sandylands, Main Street, Shawell, LE17 6AG, United Kingdom; e Agricultural Economics and Rural Policy Group, Wageningen University, Wageningen, the Netherlands

**Keywords:** Global foresight study, CGE modelling, SDGs, Bioeconomy

## Abstract

This paper takes three global visions of world development to 2050 and quantifies their implications for sustainable progress employing the metrics of the Sustainable Development Goals (SDGs). The SDG outcomes are structured through the interconnectivities of the three ‘wedding cake’ layers of ‘economy’, ‘society’ and ‘biosphere’, as posited by the Stockholm Resilience Centre. The key policy contribution is to quantify the resulting SDG synergies and trade-offs, whilst also decomposing and calculating the part-worth of the market drivers which contribute to these outcomes. The paper employs a global economic simulation model that combines rational market behaviour with environmental constraints (MAGNET) and is further extended with an SDG metrics module. A ‘non-sustainable’ world reveals trade-offs between economy and biosphere SDGs, with population growth of particular concern to a safe planetary operating space in the world’s poorest regions. Sustainable visions could reduce natural resource pressures and emissions and meet energy requirements at potentially limited economic cost. Notwithstanding, these futures do not address income inequalities and potentially increase food security concerns for the most vulnerable members of society. Consequently, developed region led international cooperation and in-kind income transfers to developing countries, constitutes a necessary prerequisite to help remedy the SDG trade-offs exhibited within the more sustainable global pathways.

## Highlights

• Three world visions to 2050 are simulated with a computable general equilibrium model.
• Sustainable development goal trade-offs and synergies are measured and their drivers.
• Global economic prosperity can be achieved within a safe planetary operating space.
• Global inequalities and food security concerns need specific attention.
• Developed country burden sharing is necessary to ensure fairer sustainable futures.

## 1 Introduction

The fundamental challenge of addressing the multiple facets of sustainability to respect our planetary boundaries is well established (e.g., FAO, 2018; IPCC, 2018). In a bid to construct a series of supporting metrics to monitor 21st century social wellbeing, the 10.13039/100004420United Nations (UN) defined eight Millennium Development Goals (MDGs) for the period 2000–2015 (United Nations, 2001). For the period 2015–2030, the multi-dimensionality of sustainability was emboldened by broadening these definitions to 17 Sustainable Development Goals (SDGs) (Allen et al., 2016) where ‘progress’ for some subcategories, is gauged in reference to a specific target (United Nations, 2015). The SDG framework explicitly acknowledges interlinkages between different metrics, whilst national governments are given leeway to reconcile the SDG framework with internal implementation plans by setting national targets as well as complementing the official UN monitoring list with additional region specific definitions (United Nations, 2015).

According to Folke et al. (2016) the conceptual ‘wedding cake’ paradigm organised into three layers offers a framework for formalising the embedded interlinkages between the categories of SDGs. More specifically, the layers postulate bidirectional interconnectivities between the ‘economy’ (top of the cake) that serves the needs of ‘society’, which in turn operates within the ‘biosphere’ (bottom of the cake).^1^ The biosphere serves as the “foundation upon which prosperity and development ultimately rest” (Folke et al., 2016, pp4). A key thesis of their model that is central to the current study, is that socially desirable progress toward the 17 SDG targets, distributed across the three layers, is explicitly linked to healthy human diets and the strength of the food system.^2^

The SDGs increasingly constitute the common language of global impact assessment (Schmidt-Traub et al., 2017). Consequently, there is a need for quantitative simulation modelling assessments to deepen our understanding of the potential medium- to long-term benefits and risks associated with alternate narratives of human development. In a review of 80 simulation models grouped into eight broad methodological classes, Allen et al. (2016) evaluate such modelling capacity to support SDG planning. In their screening process, the authors assess whether the models are ‘integrated’ and ‘policy relevant’. The first criterion examined whether the model captured variables explaining an interdisciplinary selection of sustainability metrics. The second criterion assessed whether the model was dynamic for a period of up to 15 years (i.e., dealt with structural change over time), was applicable to national scale impact assessment and exhibited a demonstrated track record in this field of analysis. Of the 80 models, only eight simulation models, classified into three methodological groupings, satisfied both these criteria.^3^ This study employs one of the models identified in the shortlist of Allen et al. (2016), namely the top-down global computable general equilibrium (CGE) model known as the Modular Applied General Equilibrium Tool (MAGNET, Woltjer and Kuiper, 2014).^4^

Global CGE models include multiple economic activities and commodities that cover the whole economy and employ mathematical representations of economic theory to characterise the behaviour of consumers and producers. The CGE model is built upon national accounts databases which balance supply and demand in each economy such that all markets are in equilibrium. Global CGE models connect these national accounts datasets with detailed gross bilateral trade data flows. A trawl of the relevant literature provides only two previous *ex ante* CGE assessments of future SDG trends, trade-offs and synergies (Campagnolo et al., 2018; Moyer and Bohl, 2019). Both studies also model stylised narratives within their alternate transition pathways. In Campagnolo et al. (2018), the focus is the development of a single composite measure of sustainable development reflecting a cross section of the SDG targets. This index is subsequently designed to provide a ranking table for a selection of countries in each of their narratives. Moyer and Bohl (2019) focus on specific SDGs metrics associated with the domains of health, education and poverty, although environmental considerations are absent. Both papers offer valuable insights, although neither paper explicitly addresses the synergies and trade-offs between specific SDG measures representative of all the broad axes of sustainable development, nor do they seek to precisely calculate the relative magnitudes exerted by the market drivers that contribute to each of these outcomes.

As a result, the key aim of this study is the simultaneous measurement and quantification of the synergies and trade-offs between specific SDGs within the three broad layers of the wedding cake. Taking a time frame to 2050, our study fashions three global transition pathways, each with their economic, biophysical^5^ and demographic assumptions and firmly rooted within the current climate and energy debate. This paper offers two clear methodological advances compared with the two aforementioned CGE studies. Firstly, the study recognises the central importance of the agri-food system to the SDGs (Folke et al., 2016) and the ‘bioeconomy’ within which the agrifood sectors operate and compete for scarce resources (i.e., land and biomass) (European Commission, 2018). In this sense, amongst economy wide simulation models, MAGNET has unrivalled coverage of bio-based activities that enables a more comprehensive internalisation of the competing food/feed/energy/material uses of biomass and the associated repercussions for land use/availability within each narrative (van Meijl et al., 2018; Philippidis et al., 2019a, 2019b). Secondly, to further our understanding of the driving forces of the SDGs, the paper decomposes and measures the precise impact of each key driver (e.g., GDP, population, labour force, energy efficiencies etc.) on SDG outcomes and their contribution to the synergies and trade-offs, both within a single transition pathway and across different transition pathways.

The rest of the article is structured as follows. Section two discusses the methodology and section three presents the results under the rubric of economy-society-biosphere. Finally, section four provides some discussion and section 5 concludes.

## 2 Methodology^6^

### 2.1 Database and model

With a benchmark year of 2011, version nine of the standard Global Trade Analysis Project (GTAP) database cover 57 activities and 140 regions of the world. (Aguiar et al., 2016). The database provides national accounts data with information on the values of input demands for 57 productive activities and finished product purchases by households and the government for 57 commodities, at pre- and post-tax prices. All economies are interconnected with detailed gross bilateral trade value flows including transport margins, import tariffs and export subsidies. The MAGNET version of the GTAP database greatly extends the treatment of bio-based activity and the repercussions for the agrifood system which supports the SDGs. More specifically, this database includes additional activities covering additional biomass sources (i.e., residues, pellets, energy crops) and uses (bioenergy types and bioindustry). A fuller discussion of the MAGNET version of the database is discussed in the supplementary materials document.

**Fig. 1:**
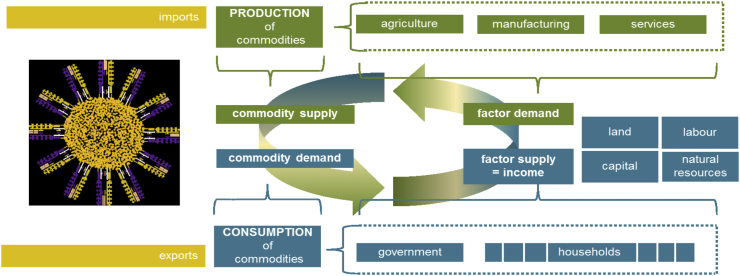
A graphical representation of the CGE model framework.

In tandem with the GTAP database, an accompanying GTAP multiregional CGE simulation model is available (Corong et al., 2017), which forms the core of the MAGNET model (Woltjer and Kuiper, 2014).^7^ As is typical of this class of model, it combines neoclassical economic theory with mathematical functional forms to represent the behaviour of producers and consumers within the closed macroeconomic system (see Fig. 1). Thus, factor demands are subject to the minimisation of costs subject to constant returns to scale production technologies, whilst the average consumer optimises utility (i.e., welfare) subject to an expenditure constraint. In each case, the structure of production technologies and consumer preferences is parameterised to characterise the price responsiveness of agents’ decisions to changing market conditions. Further market clearing equations for each market ‘i’ and accounting equations enforce the underlying ‘general equilibrium’ conditions of the model database. Thus, demand and supply in each market ‘i’ balance, ‘economic’ profits for each activity ‘j’ remain zero and the total macroeconomic value of output, income and expenditure are equal.

Built around the GTAP model, the MAGNET model employs binary switches in the model code to activate a series of non-standard modelling enhancements, which are described below. Thus, this version of MAGNET includes agricultural factor market rigidities (i.e., imperfect land transfer between agricultural activities, imperfect capital and labour mobility to/from agriculture); sustainable upper thresholds on available land and residue supplies; bioenergy mandates for conventional and advanced generation biofuels (Banse et al., 2011; van Meijl et al., 2018) and an environmental policy module to impose exogenous carbon taxes and environmental emissions limits (Burniaux and Truong, 2002). A nutrition module (Rutten et al., 2013) combines satellite data based on FAO nutritive factors for different food-types with FAO food balance sheets, thereby tracking the flow of nutrients from the source of production to the consumer.

Over long-term time horizons, MAGNET copes with structural change by employing a recursive dynamic extension which connects discrete time periods to capture cumulative growth in the capital stock and technological advancements by activities. In MAGNET, long-run food demand patterns face endogenous downward adjustments to calibrated household income elasticity parameters in regions with (rapidly) rising per capita real incomes (Woltjer and Kuiper, 2014).^8^ An additional MAGNET module captures the compounded relations between changes in greenhouse gas (GHG) levels, atmospheric concentrations, temperature change deviations and ultimately, land productivity deviations under different climate scenarios. The implementation of these ‘damage functions’ follows the assumptions and functional forms adopted in the ENVISAGE model (van der Mensbrugghe, 2010, 2015). The parameterisation of the response parameters (i.e., decay rates of carbon in the atmosphere, the radiative forcing in response to CO_2_ concentrations, temperature changes in response to radiative forcing, the response of land yields to temperature changes) is taken from Roson and van der Mensbrugghe (2010). Finally, to articulate complex results in the language of international policy assessment, a newly coded MAGNET SDG Insights Module (MAGNET SIM) calculates the impacts of each scenario for a number of SDG targets. A fuller list of these targets is presented in Annex II of this paper.

### 2.2 Transition narratives and aggregation

The key source for the transition pathways is the Global Energy and Climate Outlook (GECO) to 2050 (Keramidas et al., 2018; Weitzel et al., 2019).^9^ Based on decade time intervals to 2050, the GECO combines economic drivers with energy market balances and emissions reduction trends. Three world visions are implemented: a non-sustainable transition path (’NSUS’) and two sustainable pathways, which limit temperature rises to 2° and 1.5° above pre-industrial levels by 2100 (henceforth dubbed ’SUS’ and ’SUS+’).

**Fig. 2:**
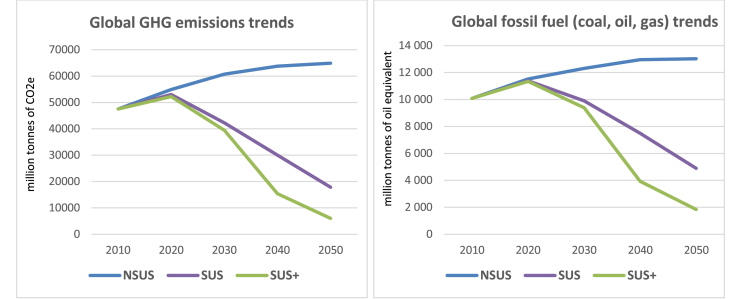
Assumed global trends in emissions and fossil energy usage. Source: own elaboration based on Keramidas et al. (2018).

The NSUS scenario assumes that progress is purely driven by market forces and technology change, with no additional climate agreements beyond 2017. More profound energy balance transition pathways (SUS and SUS+) are motivated by (i) increases in energy efficiency to decouple economic growth from energy consumption, (ii) shifting energy carriers toward electrification and (iii) decarbonisation of energy through the adoption of (bio)renewables. Fig. 2 shows the global trends for fossil energy consumption and emissions, which are implemented into our three transition pathways.

To better characterise structural economic change and allow for rapid increases in nascent bio-based technologies, the time intervals are 2011–15, 2015-20, 2020-30, 2030-40 and 2040-50. The details behind the economic, biophysical and demographic assumptions embedded within the transition pathways, are presented in Table 1. The second column shows the grouping of each of the drivers into ‘part-worths’ (Harrison et al., 2000). For example, examining the NSUS scenario results, the part-worth ‘POP’ is the cumulative change in a given SDG metric (i.e., per capita income) up to 2050, due to population change. When comparing with the NSUS scenario, the part-worth ‘POP’ is the deviation in the cumulative change in a given SDG metric up to 2050, due to population change. Taken together, the sum of the part-worths therefore equals the total cumulative change in the SDG metric for the NSUS scenario, or the total deviation in the SDG metric from the NSUS scenario.

**Table 1:** Exogenous drivers that shape the narratives of the study.

Exogenous driver	Part-worth label	Details
Region-wide productivity	GROW	Calibrated region wide productivity targeting assumed region-wide real GDP rates of change by transition pathway (Keramidas et al., 2018).
Capital stock	GROW	Changes at the same percentage rate as real GDP (fixed capital-output ratio).
Labour force	GROW	Changes at the same percentage rate as regional population (fixed long-run employment rate).
Population	POP	Exogenous rates of population change (Keramidas et al., 2018)
Carbon Tax	CT	Global increase in the carbon tax ($/tonne) by time period and transition pathway on all activities (Weitzel et al., 2019).
Energy input shifters	ENTEC	Calibrated input-output technology shifters to mimic energy balance trends by energy type and usage (Keramidas et al., 2018).
Land productivity	LNDPROD	Exogenous land productivity shocks from SSP2 pathway (Daioglou et al., 2015) plus exogenous land productivity changes calibrated to damage response functions.
Energy taste shifters	HHES	Final energy demand taste shifters to mimic transition pathway trends (Keramidas et al., 2018).
Global fossil fuel price	FUEL	Changes in fossil fuel prices by transition pathway (Keramidas et al., 2018).
Advanced bio-activity technology	BTECH	Calibrated total factor productivity technology change in advanced generation biofuels and biomaterials activities.
Biofuel mandates	BFUEL	Exogenous mandates on first-generation and advanced-generation biofuels by region.
---------	REST	A residual aggregation of drivers.

To limit the computational burden, whilst maintaining geographical and sectoral representativeness, a selection of regions is chosen to adequately capture both economic and geographical diversity across the world, whilst accommodating the explicit separation of significant key players. Thus, two large ‘developed’ country blocs (European Union (EU), USA plus Canada (USACAN)) are joined by rapidly emerging countries, China, Brazil, Russia and India. The remaining regions are Asia, North Africa, Sub Saharan Africa (SSAfrica), Latin America (LatAme), the Middle East, the Rest of Europe (REurope) and Oceania.

In terms of the sectors, the importance of the agri-food system to the successful implementation of the SDG targets postulated in the wedding cake approach, requires a detailed representation of agri-food commodities, whilst disaggregated fertiliser and feed activities allow a flexible treatment of the technological characteristics of crop and livestock production. Further sector splits support additional sources of biomass and bio-based energy and industrial applications (Banse et al., 2008; van Meijl et al., 2018). To capture the energy balance trends prescribed in Keramidas et al. (2018), fossil fuel and electricity generation activities are also represented. Finally, a number of non bio-based sectors are chosen, which either act as key blending or processing activities for elaborated bio-based inputs (i.e., chemicals, petroleum, food services) or capture logically categorised residual activities (i.e., manufacturing, services, transport) that close the macroeconomic systems in each of the regions.

## 3 Results

### 3.1 Comparing transition pathways - the big picture

**Fig. 3:**
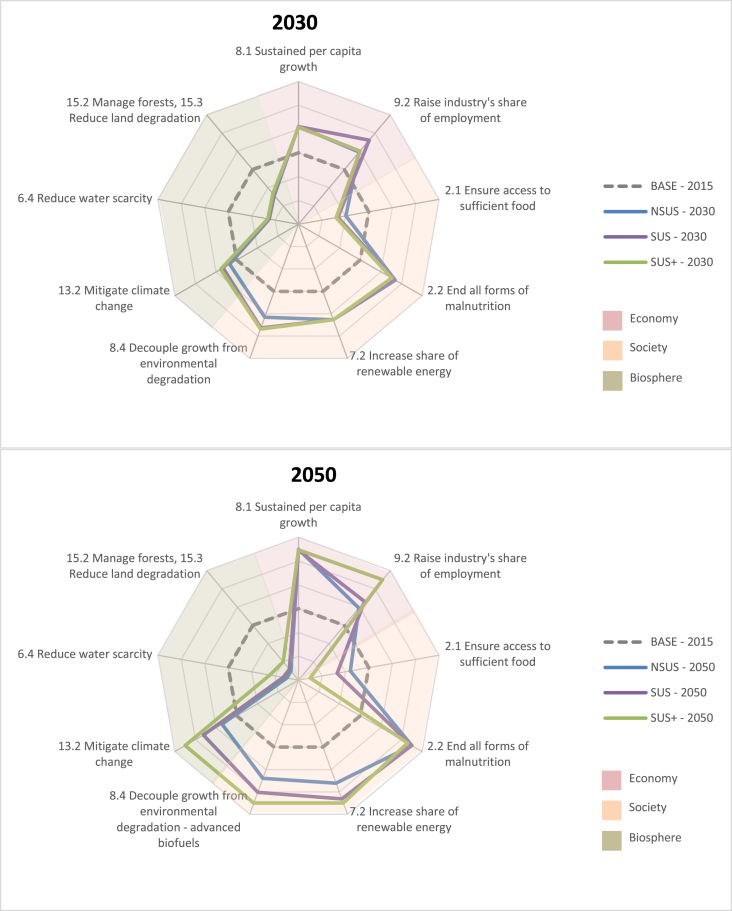
The global SDG trade-offs and synergies at a glance.

In Fig. 3 the transition pathway outcomes are compared directly for 2050. The grouping of results and the more detailed subsequent discussion follows the mapping of our SDGs to the three layers of the wedding cake model. A full set of definitions of all the SDG targets we treat here is provided in the annex II of this paper. Thus, we treat ‘economy’ (segment coloured in red) using the SDG structural indicators of per capita (pc) real incomes and employment. ‘Society’ (segment coloured in orange) and its wellbeing is reflected by the SDG metrics of food security/nutrition and (bio-) renewable energy market developments. Finally, the ‘biosphere’ layer (segment coloured in green) is represented by the trends in emissions and agricultural land and irrigation water use.

Fig. 3 presents world-wide estimates for 2030 and 2050, although the apparent trends are also valid for many regions in the study. Table 2 provides the precise values for these SDG targets that accompany the figures. The range of each spine in Fig. 3 is determined by the outcomes for each indicator across the three scenarios in 2030 and 2050. The indicator value for each scenario is located along the spine according to their position within the range. To visualise the results, the dashed circular line represents the year 2015 at zero, whilst NSUS, SUS and SUS+ are marked as blue, purple and green lines respectively. An inward movement away from the 2015 base line is a regression away from the Goal (i.e., less desirable) and an outward movement shows progression towards the Goal.^10^

**Table 2:**
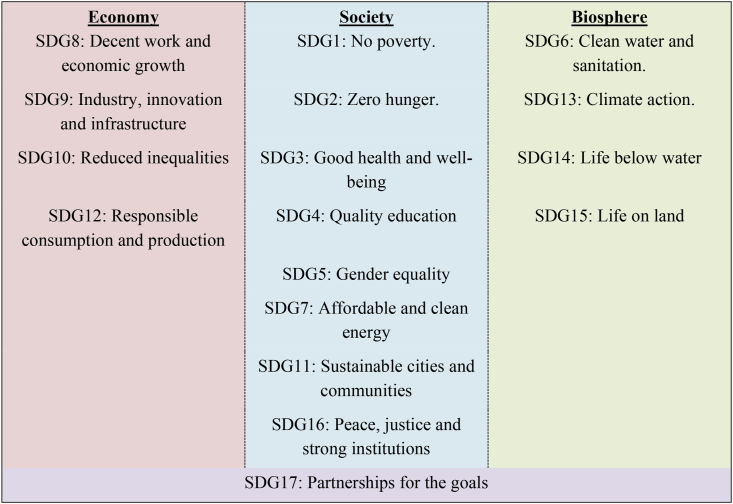
Global progression towards, or regression away from, selected SDG targets.

A cursory glance at Fig. 3 reveals that the trends exhibited in 2050 along each of the axes reflect continuations of the tendencies recorded for 2030. Comparing with the NSUS, greater planetary responsibility in SUS and SUS+, bought about by deeper transformations in energy markets and emissions cuts, attracts clear synergies between SDG metrics relating to the biosphere (SDG targets 13.2 for Climate Action and 15.2 for Life on Land) and the renewable energy markets within the layer of society (SDG target 7.2 for Clean Energy).

On the other hand, even by 2050, fossil energy usage is not completely decoupled from economic performance, which implies a trade-off. In our analysis, these trade-offs are referenced in terms of the impacts on food security (i.e., food prices, calorie intake) relating to SDGs 2.1 and 2.2, as well as macroeconomic growth (SDG 8.1), where by 2050, SUS and SUS+ outcomes are clearly inferior to that of the NSUS, although interestingly, the world average food security cost of the SUS+ is relatively limited when put into the context of the associated emissions reductions. The employment trends are rather more ambiguous and result from explainable (sometimes regional specific) factors within the analysis. To further enrich this broad picture, the following three sections drill down further into some of the results.

### 3.2 Economy

As a principle market driver, heterogeneous projected rates of real GDP are driven by region-wide productivity changes and primary factor (i.e., labour and capital) growth (GROW). Subject to the macroeconomic accounting conventions of the model, output changes generate matching changes in nominal incomes and expenditures. In the context of the SDGs, the assumed GDP projections for the poorest regions do not match the desired annual rates of 7%, as prescribed in target SDG 8.1 (see annex II). For example, with the highest per annum projected growth rates, Sub Saharan African per annum growth is still only expected to average 6.3%. Pairing the GROW and regional population growth (POP) drivers, determines structural change and prices, from which the model calculates the impacts on real pc (net) national income (in euros).

**Fig. 4:**
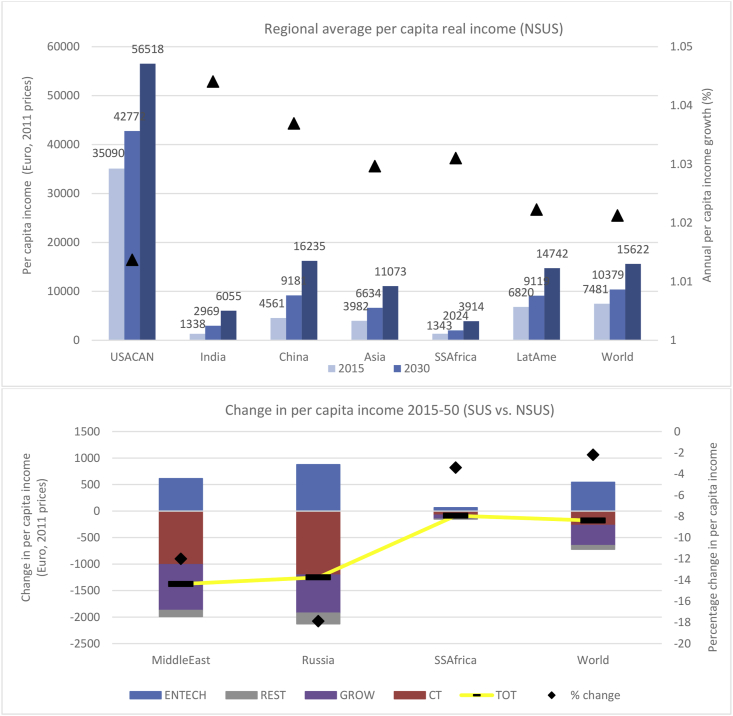
Changes in per capita real income in the NSUS pathway (upper panel) and comparing between the NSUS and SUS pathways (lower panel).

Normalising the trends reported in the upper panel of Fig. 4 by the world average, reveals evidence of real pc income convergence (SDG 10) in the NSUS pathway, although in poorer world regions, this is occurring at a glacially slow pace. As a multiple of the world average real pc income, in the USACAN, falls from 4.7 in 2015 to 3.6 by 2050. In Latin America, real pc income convergence is dampened by high expected rates of population growth, whilst in Asia, real pc incomes rise to 0.71 of the world average (€11,073) by 2050. Indeed, taking the case of China, with annual GDP growth of 4.4% and a slight fall in the population expected by 2050, real pc income is almost at parity with the world average by 2040 (not shown), and surpasses it comfortably by 2050. In the Sub Saharan Africa, a threefold rise in real pc incomes over the 35-year period to 2050 is expected (€1,343 in 2015; €2,024 in 2030; €3,914 in 2050). Notwithstanding, as in India, fast paced economic progress is stifled by a more than doubling of Sub Saharan Africa’s population. Thus, Sub Saharan African average wealth rises from 0.21 of the world average in 2015 to only 0.30 by 2050.

In the lower panel of Fig. 4, the black bars in the yellow line (TOT) show the total cumulative deviation (in million euros) by 2050 in real pc income comparing the SUS with the NSUS pathway. The contribution of the part-worths of each driver (see Table 1) to this deviation clearly highlight the synergies and trade-offs with per capita incomes. As noted in section 3.1, there is evidence of the trade-off between planetary responsibility and economic progress, where the black dot shows a negative marginal impact on average world pc real income (−2.2%), which is even more marked in the poorest region of the world (Sub Saharan Africa), and the fossil-based economies of the Middle East and Russia.

Examining the drivers of the deviations (SUS vs NSUS) in the lower panel of Fig. 4, investment driven energy efficiency gains (ENTECH) drive increasing economic rents to the production factors, leading to rising real pc income. On the other hand, the extent to which fossil energy usage remains coupled to economic progress is also clearly visible. Indeed, higher carbon taxes (CT) raise input and product prices on emitting activities, which depresses real pc incomes. The marginal impact of the GROW driver is negative which highlights continued (albeit limited) coupling between fossil energy dependence and economic growth even by 2050 (SDG 8.4). For example, in the fossil fuel exporting regions of Russia and Middle East, real pc income in the SUS scenario falls −18% (-€1,251 pc) and −12% (-€1,376 pc), respectively. This is strongly driven by the CT driver, which depresses real incomes -€1,206 pc and -€1,010 pc in Russia and the Middle East, respectively. Sub Saharan Africa also faces higher than global average pc income reductions of −3.4% (-€87 pc), which are again driven by ENTECH (€64 pc), CT (-€75 pc) and GROW (-€75 pc) effects. Importantly, in neither of the alternate sustainable transition pathways, is there any noticeable change in pc real income inequalities by 2050 (SDG 10). In fact, normalising by the global average, the gap between the wealthiest (USACAN) and the poorest (Sub Saharan Africa) actually widens very slightly in both the SUS and SUS+ scenarios.^11^

Examining structural changes from the barometer of full and productive employment (SDG 8.5), there is a haemorrhaging of global agricultural labour, from just over 1.145 billion in 2015 to 1.003 billion in 2050, with pronounced falls in China and India (19% and 35%, respectively). In Sub Saharan Africa, agricultural employment rises from 171 million in 2015, to a peak of 209 million persons in 2040, before dropping back to 196 million in 2050. This ‘n’ shape trend reflects the opposing forces of rapid growth and demand for food coupled with a strengthening trend of structural change arising from rapid industrialisation.

**Fig. 5:**
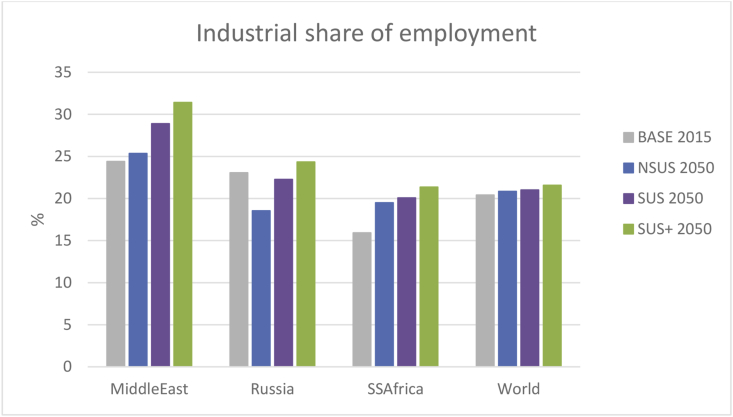
The industrial share of employment in 2015 and 2050 in the transition pathways.

As a consequence of the agricultural migratory outflows observed above, in the NSUS pathway the industrial share of employment worldwide (SDG 9.2) rises, but only slightly, from 20.4% in 2015 to 20.8% by 2050 (Fig. 5). In particular, the Asian continent continues its drive as the workshop of the world with a rising industrial share of employment from 19.8% in 2015 to 21.6% by 2050. Similarly, in Sub Saharan Africa, the rise in this share statistic is also accentuated (15.9% in 2015, 17.5% in 2030 and 19.5% by 2050), but apparently well short of the suggested doubling of the share target highlighted in SDG 9.2.

Comparing with the NSUS pathway, the SUS pathway retains higher agricultural employment by 2050 because relatively slower macroeconomic growth reduces the pace of structural change, with the result that agricultural migration to industrialised and service sectors is more muted. In the SUS+ scenario the effect on the agricultural workforce is reversed by the negative production impact of the CT driver on emissions intensive agricultural activities, such that the primary agricultural labour exodus by 2050 is accelerated (not shown). In both SUS and SUS+ pathways, the global industrial share of employment rises only slightly compared with the NSUS (20.8% in NSUS, 21.0% in SUS, 21.6% in SUS+) (Fig. 5), whilst the Middle East, Russia and Sub Saharan Africa exhibit above global average progress toward the target of doubling the industrial share of employment, although still some way from being achieved.

### 3.3 Society

Related to poverty and food security, is food accessibility through affordability (SDG 2.1). For many regions food prices remain relatively stable in the NSUS, SUS and SUS+ pathways, as productivity growth on production and land help to mitigate population and income rises. In Sub Saharan Africa, by 2050 there are important expected improvements in total factor productivity and land productivity (LNDPROD) in the NSUS scenario. In isolation, these factors reduce the food costs index (2011 = 100) by 32% and 15%, respectively (not shown). At face value, this is clearly a positive result, although caution should be exercised as these trends are dependent on ‘expectations’ of technological growth, and should not be taken as clear indicators on food affordability for the poorest segments of society, but rather for the average Sub Saharan African citizen. Moreover, based on the same food cost index, the results also reveal the consequences of unchecked POP growth on this continent which increases food costs by 27.8 points (not shown).

Unfortunately, planetary responsibility characterised by the SUS and SUS+ pathways brings detrimental impacts on food costs. By 2050, the marginal rise in the global food price index compared with the NSUS pathway is 1.2% (SUS) and 3.6% (SUS+), whilst all developing regions are worse affected, with food price rises in Sub Saharan Africa of up to 8.4% (SUS+). Clearly, from a food security standpoint, even slight food price rises are particularly concerning for individuals living in vulnerable areas. The CT driver is key here, which impacts most on more emissions intensive agricultural sectors in developing regions.

**Fig. 6:**
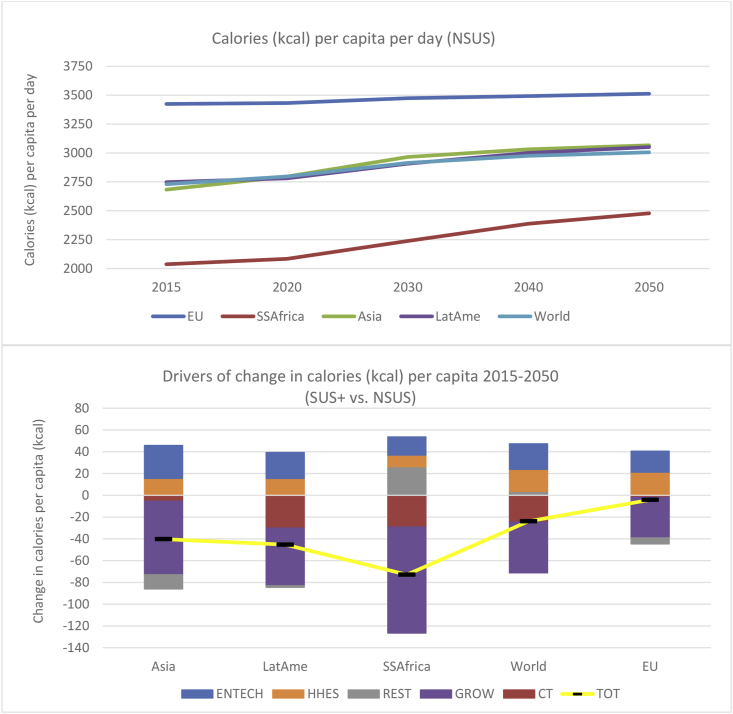
The evolution of calorie intake in the NSUS pathway (upper panel) and the trade-offs comparing with the SUS+ scenario (lower panel).

Linked to the above is available kilocalorie (kcal) intake on a per capita per day (pcpd) basis, which forms a proxy for malnutrition (SDG 2.2). Fig. 6 shows the evolution of gross calorific content of food available for consumption by households, although this overstates ‘actual’ calorie consumption since it does not account for heterogeneous regional rates of waste along the supply chain. Indeed, taking only the household part of the food chain, FAO (2011) reports that food waste rates of total consumption can vary significantly between wealthier countries (20–30% in North America and the EU) and poorer regions (1–2% in Sub Saharan Africa).

Encouragingly, for the 35-year period to 2050, the upper panel of Fig. 6 shows steeper increases in pcpd calorie intake in relatively poorer regions. World average pcpd calorie consumption rises 10%, or 276 kcal pcpd, from 2,730 kcal pcpd in 2015 to 3,006 kcal pcpd in 2050. Over the same period, EU consumption rises only 2.8% from a total of 3,424 kcal pcpd (88 calories pcpd), whilst in Sub Saharan Africa, from a starting value of 2,037 kcal pcpd, the corresponding rises are 22% and 441 kcal pcpd. A comparison of our NSUS with a business as usual scenario in FAO (2018, pp104), reveals that both studies expectations of pcpd calorie intake are quite closely aligned. In our study, rising calorie intake is slightly higher since our real GDP assumptions are more optimistic, whilst assumed population increases in the FAO (2018) study are stronger.

In the lower panel of Fig. 6, the key drivers in the deviation of calorie demand from the NSUS pathway are rising incomes associated with economic growth, improvements in land productivity and population change (not shown). In Sub Saharan Africa, for example, expected rapid changes in the first two factors are linked with 710 kcal pcpd and 29 kcal pcpd increases, respectively, whilst population growth depresses pcpd calorie intake by 304 kcal.

Comparing with the NSUS pathway, in both the SUS and SUS+ scenario, calorie intake falls, although this negative impact is far more pronounced in the SUS+ scenario (lower panel, Fig. 6). By 2050 the global average human pcpd consumption falls −24 kcal, whilst in Latin America and Sub Saharan Africa, this statistic falls by −45 and −73 kcal pcpd, respectively. This deviation from the NSUS is linked to an income effect (slower pc income rises) and a price effect (strong rises in food costs from the CT driver). Partially offsetting these drivers are kcal pcpd rises driven by energy efficiency gains (ENTEC) and expenditure savings in residential energy usage (HHES).^12^ It should be noted that around the average ‘gross’ calorie statistic presented here, there are inevitably more vulnerable sections of society which would be hit considerably harder. In contrast to our study, a more sustainable scenario in FAO (2018) indicates a calorie intake rise in many regions compared with their business as usual scenario. Given the strength of the CT driver reported here, this apparent contradiction is possibly reconciled by the fact that their sustainable scenario allows temperature rises of up to three degrees above pre-industrial levels by 2100.

In ensuring reliable access (SDG 7.1) and sustainable energy sources (SDG 7.2), for electricity generation, even the NSUS scenario envisages important changes driven by rapid acceleration of solar and wind turbine technologies, although exclusively as a response to natural resource depletion and rising fossil prices. Based on quantities in million tonnes of oil equivalent (mtoe), global renewable^13^ electricity generation has a volume share of 12% in 2015, rising to 51% by 2050 (not shown). An interesting outlier is Brazil, with its plentiful access to hydroelectric sources, is already 80% renewables based in 2015, rising to 97% by 2050 (not shown). Dominated by wind and solar energy sources, in the sustainable transition pathways the global renewable output volume share of electrical energy generation in 2050 rises from 51% (NSUS), to 75% (SUS) to 86% (SUS+). The global share of electricity generation from biomass rises from 0.5% (NSUS), to 1.2% (SUS) to 2.1% (SUS+) (not shown).

**Fig. 7:**
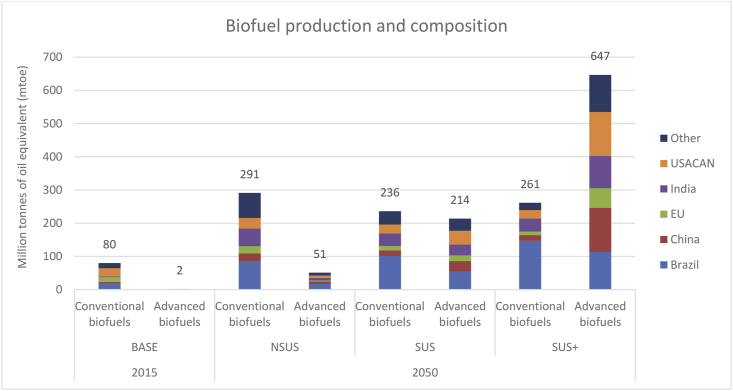
The global quantity and regional composition of conventional and advanced biofuels production in the transition pathways.

Accompanying the sustainable transition pathways is the switch in liquid biofuels toward advanced generation biofuels based on non-food lignocellulosic feedstocks (i.e., miscanthus, switchgrass) and crop/forestry residues. Fig. 7 shows that global capacity of conventional biofuels in the NSUS rises from 80 mtoe in 2015 to 291 mtoe by 2050. Encouragingly, examining the individual market drivers, there is little evidence of strong food price rises arising from increased conventional biofuels production in any of the transition pathways. With its competitive edge due to the abundance of its land endowment and the more sustainable nature of ethanol production, Brazil’s share rises from one-quarter in 2015, to 30% by 2050 in the NSUS pathway (Fig. 7).

In the absence of clear sustainability directives in the NSUS, the advanced biofuels market remains small by comparison, rising from 2 mtoe in 2015, to 51 mtoe by 2050. One-third of this production in 2050 corresponds to Brazil. As Fig. 7 reveals, world conventional biofuels production remains fairly static across the three scenarios. On the other hand, with a drive toward sustainable energy, by 2050 the combined conventional and advanced liquid biofuel market grows from 342 mtoe in the NSUS, to 450 mtoe in SUS, to 908 mtoe in SUS+.

### 3.4 Biosphere

**Fig. 8:**
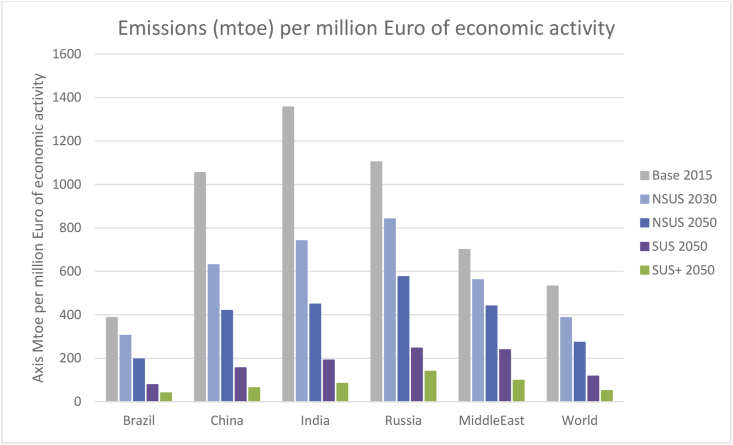
Decoupling economic activity from emissions.

In reference to SDG 13.2, which integrates climate change measures into national policies, strategies and planning, the NSUS pathway reveals clearly negative trends as global greenhouse gases rise 26% compared to 2015. On the other hand, there is a ‘decoupling’ or loosening of the positive correlation between economic growth and GHG emissions. For example, in million tonnes of CO_2_ equivalent per million euros of economic activity (mtCO_2_e/m€), Fig. 8 shows global emissions falling from 532 mtCO_2_e/m€ in 2015, to 273 mtCO_2_e/m€ in 2050. In rapidly growing economies such as India and China, there is more rapid convergence to this global average. In contrast, the fossil rich economies of the Middle East exhibit slower falls (700 mtCO_2_e/m€ in 2011 to 440 mtCO_2_e/m€ in 2050).

In the SUS and SUS+ narratives, global GHG emissions are 59% and 79% below the NSUS pathway. Thus, compared with the 2050 NSUS pathway value of 273 mtCO_2_e/m€, the SUS and SUS+ pathways record corresponding values of 117 mtCO_2_e/m€ and 51 mtCO_2_e/m€, respectively (Fig. 8).

As a proxy for sustainable land management consistent with SDGs 15.2 and 15.3, changing patterns of agricultural land are reported. In the NSUS pathway, global agricultural land of 4.85 billion ha in 2015, rises 7.4% (360 million ha) by 2050, equivalent to twice the EU’s agricultural land area in 2015. Of this increase, global cropland rises 12.6% (184 million ha)^14^ and pastureland rises 5.2% (176 million ha) (not shown).^15^

**Fig. 9:**
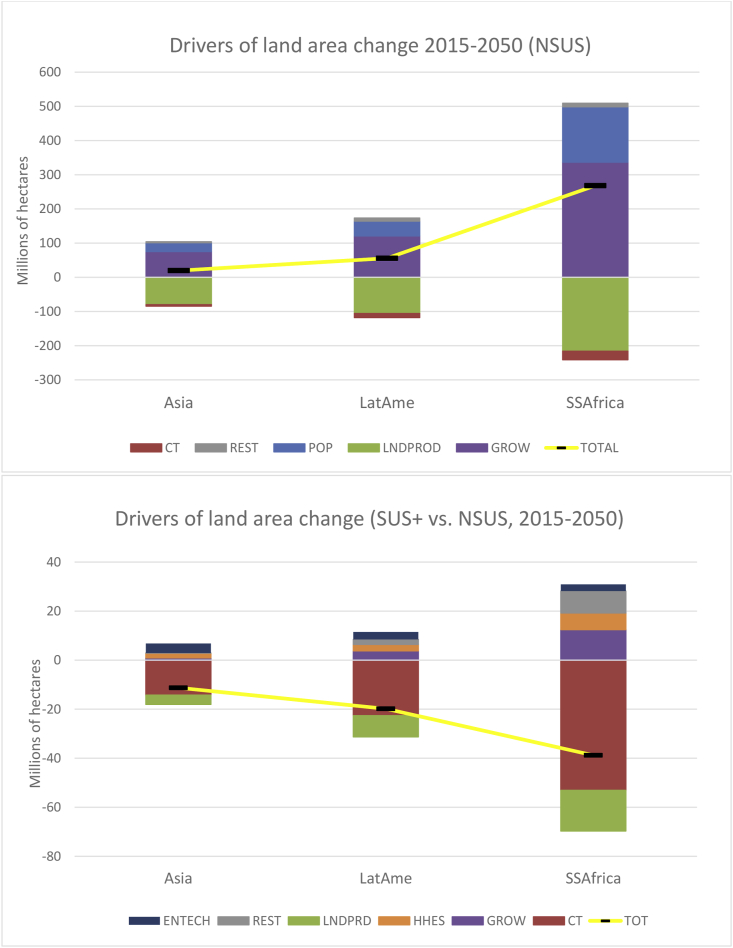
The driving forces of agricultural land usage in the NSUS pathway (upper panel) and comparing between the NSUS and SUS pathways (lower panel).

Across the regions, Fig. 9 shows that rising global agricultural land uptake in the NSUS pathway is dominated by Sub Saharan Africa (268 million ha, or 25% increase) and, to a lesser extent, Latin America (56 million ha, 7.5% increase). In all regions, GROW and POP are key drivers (top panel in Fig. 9). In Sub Saharan Africa, for example, these drivers account for 337 million ha and 162 million ha, respectively. In all regions, these land increasing effects are partially offset by land saving (bio)technology adoption and innovation, which in Sub Saharan Africa is estimated to save 216 million ha of agricultural land over the 35 year time period (upper panel Fig. 9).

Comparing with the NSUS pathway, a synergy between SUS and SUS+ pathways and reduced agricultural land usage, is clearly observable. Global agricultural land savings are between 15 million ha (SUS) and 74 million ha (SUS+), equivalent to approximately 8% and 42%, respectively, of the EU’s current agricultural land area. The effect the CT driver on dampening food demand, subsequently saves up to 99 million ha (SUS+) of global agricultural land, largely affecting Sub Saharan Africa (53 million ha) (lower panel Fig. 9). In addition, LNDPROD improvements due to reduced radiative forcing from reduced temperature increases also has a land saving effect of up to 35 million ha worldwide (approximately 20% of the EU’s agricultural land area), with land saving gains distributed to all regions (lower panel, Fig. 9).^16^

Abstracted irrigated water consumption is a direct function of changes in crop land areas, such that the story of the underlying drivers is the same as that for land. In the NSUS pathway, the associated rise in global irrigated (blue) water volume abstraction over the 35 year period is 128 billion cubic metres (m3) of water or 4.3% (not shown). This rise is equivalent to approximately the irrigated water requirement of the entire North African region in 2050.

**Fig. 10:**
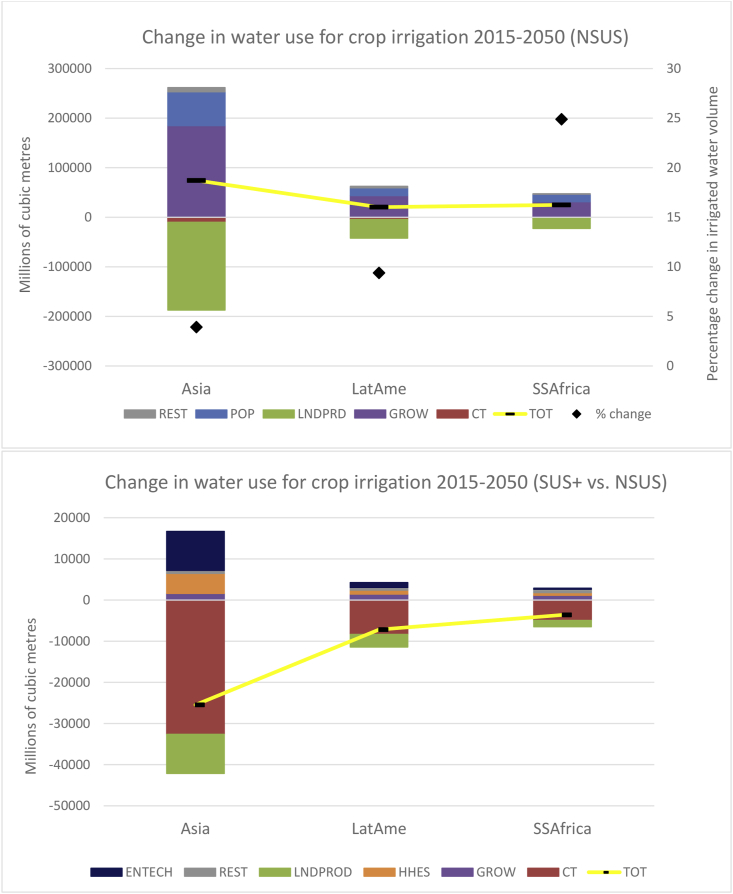
The driving forces of water usage for crop irrigation in the NSUS pathway (upper panel) and comparing between the NSUS and SUS+ pathways (lower panel).

The global increase in abstracted irrigated water in the NSUS scenario is largely driven by Asia (upper panel of Fig. 10), with particular emphasis on rice production. The GROW and POP drivers in Asia, account for 185 billion m^3^ and 68 billion m^3^ of irrigated water abstraction, respectively (Fig. 10). In water scarce regions of the world such as Sub Saharan Africa, the rise in abstracted water volume is 24% (black dot in upper panel of Fig. 9), again motivated by GROW and POP drivers. As in the case of land use, the essential role of (bio)technology is key, which worldwide is estimated to save 250 billion m^3^ of abstracted irrigated water.

Comparing with the NSUS in 2050, there are marginal savings of 7.9 billion m^3^ and 37.6 billion m^3^ in the SUS and SUS+ pathways, respectively. Focusing on the SUS+ scenario (lower panel of Fig. 10), the pattern of market drivers behind these deviations is identical to that of land, although the bulk of the water savings emanate from Asia. The CT driver saves 48.5 billion m^3^ of water worldwide (32.6 billion m^3^ from Asia), whilst limiting temperature rises and the resulting LNDPROD improvement saves close to 15.8 billion m^3^ of water worldwide (9.5 billion m^3^ from Asia).

## 4 Discussion

Within the framework of the Sustainable Development Goals (SDGs) set out by the United Nations (UN), national governments are expected to plot their own roadmaps to achieve socially desirable targets of human development. Taking a global perspective, the wedding cake paradigm (Folke et al., 2016) embeds the SDGs within the three ‘layers’ of economy, society and biosphere, positing bi-directional interconnectivities between them, thereby enabling integrated decision making. The mobilisation of this interconnected approach into policymaking is already evident in the EU’s Green Deal, where the aim of establishing a socially fair transition places the SDGs at the heart of concerted EU action (European Commission, 2019).

In a non-sustainable (NSUS) world where engagement by society is limited to economic necessity rather than planetary responsibility, this paper shows evidence of SDG synergies (e.g., per capita income (SDG 8.1), industrial share of employment (SDG 9.2), calorie intake (SDG 2.2)), as well as SDG trade-offs (e.g., rising emissions (SDG 13.2), pressures on available land (SDG 15.2) and irrigated water (SDG 6.4)), which are particularly acute in the poorest regions (i.e., Sub Saharan Africa).

The paper also quantifies the degree to which macroeconomic and population growth, especially in Sub Saharan Africa, encroach on our planetary boundaries. Indeed, the finding by Bren d’Amour et al. (2017) that 80% of cropland losses to degradation and urbanisation worldwide are concentrated in Africa (and Asia), puts this result into sharp focus. In the context of population pressures, there is a clear and present need for investments in education and improved access to (*inter alia*) reproductive health services, particularly in Sub Saharan Africa, to arrest this trend (Abel et al., 2016; Bongaarts and O’Neill, 2018). Beyond public health concerns, they are also crucial to avoid potential humanitarian crises arising from the emergence of possible conflict zones as competition for scarce resources is intensified.

Our quantitative estimates also reveal the extent to which land productivity improvements are crucial counter drivers against rising population pressures, providing a synergy effect for realising both food security (e.g., SDG 2.1 and SDG 2.2), and responsible planetary management (e.g., SDG 6.4, SDG 13.2 and SDG 15.2). Yield improvements are, however, shrouded in uncertainty, where significant yield gaps remain between poorer and wealthier countries worldwide and yield improvements for cereals, oilseeds and sugar in the richest countries are reaching a peak (FAO, 2018). Thus, global food security fears may only be alleviated through yield improvements in poorer regions supported by both capacity building and unfettered trade access to richer country markets. Unfortunately, current evidence suggests that private agricultural research and development investments and interests remain the preserve of the higher income regions (FAO, 2018).

Sustainability pathways, driven by greater emissions cuts, deeper energy market transformations and marginal improvements for land productivities resulting from lower global temperature rises, unambiguously ease planetary pressures in terms of land usage (SDG 15.2) and water abstraction (SDG 6.4). Indeed, said non-market gains are undervalued in this paper, which are undoubtedly accompanied by additional benefits relating to improvements in ecosystem services and biodiversity (IPBES, 2019). A further synergetic effect within the sustainability pathways is the role that advanced biotech improvements can play in the energy market, through the adoption of advanced generation biofuels dependent on non-food lignocellulosic crops, which as well as meeting energy security needs (SDG 8.4), do not significantly raise food prices nor compromise food security (SDG 2.1).

Notwithstanding, as a part measure for achieving greater planetary responsibility within these sustainable pathways, higher carbon taxes exact a high food security cost in some parts of the world in terms of increasing average food prices and resulting reductions in average calorie intake. Importantly, these ‘average’ effects are highly prevalent in Sub Saharan Africa, which for the very poorest members of society could have grave implications.

This mismatch between satisfying food security and minimising environmental impacts is reported by other commentators (Berners-Lee et al., 2018). As a result, it is essential that sustainable pathways do not leave the most vulnerable behind. Once again, this calls on richer countries to collectively commit to cooperative schemes, for example, even greater burden sharing of emissions, as pledged within the Paris Agreement. These results do not negate the need for mitigation efforts, but rather highlight the need for policy designs that explicitly include complementary measures in order to avoid an increase in hunger and malnutrition (Fujimori et al., 2019). Recently, Doelman et al. (2019) showed that 9% crop productivity growth is needed to mitigate the food security effect and with low-meat diet changes this would be 7%. Alternatively, concerted action spearheaded by rich country governments and/or stakeholders (i.e., consumers) to reduce food waste may also alleviate food price rises and land use pressures (Philippidis et al., 2019b), greenhouse gas emissions (Springmann et al., 2018) and water footprints (Vanham et al., 2018). Interestingly, Berners-Lee et al. (2018) estimate that the removal of human edible crops from animal production systems, would do even more than either land yield improvements or food waste reductions to meet the world’s food security needs by 2050.

As with all simulation modelling exercises, there are caveats to be observed and areas for further research. The characterisation of technology change remains exogenous to the model, whilst a more desirable treatment would involve an endogenous internalisation of the associated costs of different research and development initiatives. To better address inequality, an explicit disaggregation of household types would provide greater insights on within-country income distributions and purchasing power differences. A more explicit treatment of the structural transition of labour skills in tandem with economic development in medium- to long term scenarios would also enhance the analysis. In the field of climate change, other mitigation options could be internalised within the modelling framework, such as carbon capture and utilisation (CCU). Moreover, whilst the MAGNET model has been extended to quantify a list of SDG metrics, additional effort, focusing on a combination of *ex-post* econometric estimates coupled with *ex-ante* simulation experiments (Costanza et al., 2016), could be directed toward extending and enhancing the treatment of non-market SDG metrics across additional domains (e.g., ensuring healthy lives, educational quality, gender equality, sustainable cities and communities or the promotion of peaceful societies).

## 5 Conclusion

This paper quantifies sustainable progress trade-offs and synergies resulting from three visions of world development to 2050. Sustainable progress, defined by the Sustainable Development Goal (SDG) targets, is mapped to the broad axes of ‘economy’, ‘society’ and ‘biosphere’ contained in the wedding cake framework. An in-depth review of simulation modelling taken from the literature supports the MAGNET computable general equilibrium model as one of a small selection of key candidates for SDG impact assessment. Moreover, an important pillar of the study is the recognition of the importance of the agri-food system to the successful implementation of the SDG targets. Once again, compared with other global CGE models, MAGNET contains an unrivalled explicit market treatment of alternate biomass sources and biomass-competing activities (e.g., food, feed, industry, energy), within which the agrifood system operates. Furthermore, to enumerate a broad selection of SDG targets, a tailor-made SDG insights module is encoded within the MAGNET modelling platform. Uniquely from a policy perspective, the study not only examines SDG synergies and trade-offs representative of all the axes of sustainability simultaneously, but also decomposes and calculates the part-worth of the market drivers which contribute to these outcomes.

As expected, a non-sustainable world order reveals trade-offs between economic and biosphere SDGs, although in the world’s poorest regions, population growth is of particular concern to a safe planetary operating space. Sustainable visions could reduce global natural resource pressures and emissions and meet energy requirements at potentially limited economic cost. Notwithstanding, these futures do not address income inequalities across the world, whilst the food security impacts could be harmful to the most vulnerable members of society. Thus, to successfully operationalise these pathways, requires in-kind income and knowledge transfers from the richer world. Accordingly, further research should examine and credibly quantify the positive expected impacts of these types of concerted actions to global society, whilst internalising the expected economic costs to the rich world. Precedents of concerted efforts on the international stage (i.e., Green revolution, HIV/AIDS, COVID19) suggest that there are grounds for optimism.

## Author contributions

George Philippidis led the study. He provided technical help with the creation of the SDG SIM module in MAGNET, conducted the modelling, ran the simulations and processed the results. He also wrote the abstract and led the writing of the paper. Lindsay Shutes led the creation of the SDG SIM module in MAGNET, was responsible for the graphical presentations and provided feedback on the paper. Tévécia Ronzon helped to steer the direction of the paper in the context of the literature and collaborated with George Philippidis on section one of the paper. Robert M’Barek provided valuable input into the conception of the research idea, helped with the motivation and design of the simulations and provided feedback on earlier drafts of the paper. Andrzej Tabeau provided important technical input into the SDG SIM module of MAGNET and provided feedback on an earlier draft paper. Hans van Meijl helped conceive the idea and the writing of sections four and five.

## Role of the funding source

This work was partially supported by the 10.13039/100010661European Union’s Horizon 2020 BioMonitor project [grant agreement N°773297], entitled “Measuring the BioEconomy”, and the from the European Commission [Administrative Arrangement N° JRC 34488-2016]. The lead author also recognises funding received from the 10.13039/501100011003Instituto Nacional de Investigación y Tecnología Agraria y Alimentaria (INIA) (RTA2017-00046-00-00), for the project “Bioeconomía 2030: Un análisis cuantitativo de las perspectivas a medio plazo en España” co-funded by FEDER.

## Declaration of competing interest

The authors declare that they have no known competing financial interests or personal relationships that could have appeared to influence the work reported in this paper.

## Figures and Tables

**Table:** 

Economy	Society	Biosphere
SDG8: Decent work and economic growth	SDG1: No poverty.	SDG6: Clean water and sanitation.
SDG9: Industry, innovation and infrastructure	SDG2: Zero hunger.	SDG13: Climate action.
SDG10: Reduced inequalities	SDG3: Good health and well-being	SDG14: Life below water
SDG12: Responsible consumption and production	SDG4: Quality education	SDG15: Life on land
	SDG5: Gender equality	
	SDG7: Affordable and clean energy	
	SDG11: Sustainable cities and communities	
	SDG16: Peace, justice and strong institutions	
SDG17: Partnerships for the goals		

SDG17 transcends all three layers of the cake (Folke et al., 2016).

**Table:** 

	SDG Target	Target Description
**Economy**	8.1	Sustain per capita economic growth in accordance with national circumstances and, in particular, at least 7 per cent gross domestic product growth per annum in the least developed countries
	8.4	Improve progressively, through 2030, global resource efficiency in consumption and production and endeavour to decouple economic growth from environmental degradation, in accordance with the 10-Year Framework of Programmes on Sustainable Consumption and Production, with developed countries taking the lead
	8.5	By 2030, achieve full and productive employment and decent work for all women and men, including for young people and persons with disabilities, and equal pay for work of equal value
	**9.2**	Promote inclusive and sustainable industrialisation and, by 2030, significantly raise industry’s share of employment and gross domestic product, in line with national circumstances, and double its share in least developed countries
**Society**	7.1	By 2030, ensure universal access to affordable, reliable and modern energy services
	7.2	By 2030, increase substantially the share of renewable energy in the global energy mix
	2.1	By 2030, end hunger and ensure access by all people, in particular the poor and people in vulnerable situations, including infants, to safe, nutritious and sufficient food all year round
	**2.2**	>By 2030, end all forms of malnutrition, including achieving, by 2025, the internationally agreed targets on stunting and wasting in children under 5 years of age, and address the nutritional needs of adolescent girls, pregnant and lactating women and older persons
**Biosphere**	6.4	By 2030, substantially increase water-use efficiency across all sectors and ensure sustainable withdrawals and supply of freshwater to address water scarcity and substantially reduce the number of people suffering from water scarcity
	13.2	Integrate climate change measures into national policies, strategies and planning
	15.2	By 2020, promote the implementation of sustainable management of all types of forests, halt deforestation, restore degraded forests and substantially increase afforestation and reforestation globally
	**15.3**	By 2030, combat desertification, restore degraded land and soil, including land affected by desertification, drought and floods, and strive to achieve a land degradation-neutral world

**Table:** 

BFUEL	Biofuel mandates (driver)
BTECH	Advanced bio-activity technology (driver)
CGE	Computable General Equilibrium model
CO_2_	Carbon dioxide
CT	Carbon Tax (driver)
ENTEC	Energy input efficiency changes (driver)
EU	European Union
FAO	Food and Agriculture Organization of the United Nations
FUEL	Global fossil fuel price (driver)
GDP	Gross Domestic Product
GECO	Global Energy and Climate Outlook
GHG	Greenhouse gas
GROW	Region-wide productivity, capital stock and the labour force growth (drivers)
HHES	Residential energy demand usage (driver)
IPBES	Intergovernmental Science-Policy Platform on Biodiversity and Ecosystem Services
IPCC	Intergovernmental Panel on Climate Change
Kcal	Kilocalorie
LatAme	Latin America
LNDPROD	Land productivity change (driver)
Million ha	Million hectares
MAGNET	Modular Applied General Equilibrium Tool
MAGNET SIM	MAGNET SDG Insights Module
MDGs	Millennium Development Goals
mtoe	Million tonnes of oil equivalent
M^3^	Cubic metres
NSUS	Non-sustainable business-as-usual transition path scenario
pc	per capita
pcpd	per capita per day
POP	Population change (driver)
REST	Residual aggregation of remaining drivers
REurope	Rest of Europe
SDG	Sustainable Development Goal
SSAfrica	Sub Saharan Africa
SSP2	Shared Socioeconomic Pathways, “middle of the road” scenario
SUS	Sustainable pathway scenario, which limits temperature rises to 2° above pre-industrial levels by 2100
SUS+	Sustainable pathway scenario, which limits temperature rises to 1.5° above pre-industrial levels by 2100
UN	United Nations
USACAN	USA plus Canada

## Footnotes

1 The biosphere is the global ecological system that integrates all living things and their interrelationships, as well as their interaction with the atmosphere, water cycle, biogeochemical cycles and the dynamics of the Earth system as a whole (Folke et al., 2016).

2 In the annex I, the categorisation of the SDGs into the three layers of the wedding cake model is defined.

3 The three model classes were: top-down computable general equilibrium; a system dynamics model and six hybrid approaches based on a platform of model links. Please see Allen et al. (2016) for a further discussion of the model typologies.

4 Employing the term from Allen et al. (2016), ‘top-down’ refers to the spatial dimension of a model with (typically) macroeconomic or economy-wide coverage. They are used for answering national scale questions across a range of aggregated economic activities.

5 This refers to the physical input-output conversion factors embedded within the model assumptions. In particular, we are talking about the productivity of land in agriculture and the available supplies of agricultural land and residues as sources of biomass provision to the agrifood system and the bioeconomy. These assumptions are taken from the biophysical land use model IMAGE (Daioglou et al., 2015).

6 A supplementary materials document provides additional discussion of CGE models, the MAGNET version of the GTAP database, the MAGNET SDG insights module, discussion of the narratives, the regional and sectorial aggregation, and the CGE modelling assumptions and calibration steps. This document also provides additional information on the presentation of the results

7 This paper describes the key features of MAGNET used for this study. The interested reader can access a full documentation of the model from https://www.magnet-model.org/.

8 This modification permits a more realistic (i.e., moderate) increase in food demand in regions undergoing rapid economic development (i.e., Africa, Latin America, China, India). Compared with the standard GTAP model, this module moderates upward pressure on the prices of land, biomass and ultimately, food.

9 Full details are available online from Keramidas et al. (2018).

10 The precise SDG scaling in Fig. 3 is detailed in the supplementary materials document (see S.5.1).

11 By 2050, Sub Saharan African per capita real income as a proportion of the global average is 0.251 (NSUS), 0.248 (SUS) and 0.242 (SUS+). For the USACAN, the corresponding statistics are 3.618 (NSUS), 3.633 (SUS) and 3.656 (SUS+).

12 Despite marginal real income losses as noted above, in poorer countries, the household expenditure savings on energy costs are largely transferred to increasing the food budget expenditure share.

13 Renewables are defined in terms of solar, wind, hydroelectric and biomass.

14 This estimate is compared with a ‘business as usual’ increase of 11% in global arable land (165 million ha) recorded by FAO (2018) from 2012 to 2050.

15 These increases are equivalent to 0.34% and 0.14% per annum percentage rises in arable- and pasture-land, respectively. This compares with corresponding per annum global rates of growth of 0.27% and 0.5% over the 1962–2015 period (Chini et al., 2019).

16 For further information, see the discussion of the damage function in the methodology section.
