# Supplementary material for: Snakes and ladders: World development pathways’ synergies and trade-offs through the lens of the Sustainable Development Goals
Source: J Clean Prod. 2020 Sep 10;267:122147. doi: 10.1016/j.jclepro.2020.122147 (PMC7323613; doi:10.1016/j.jclepro.2020.122147)
Supplement: Multimedia component 1 [file mmc1.docx]

**Supplementary material**

**S.1 Methodology**

**S.1.1 Computable General Equilibrium (CGE) Models - overview**

The Modular Applied GeNeral Equilibrium Tool (MAGNET) is a class of neoclassical multi-region, multi-commodity computable general equilibrium (CGE) economic simulation model ([Woltjer and Kuiper, 2014](#_ENREF_12)) and has an established pedigree in a number of high-profile foresight studies for international and intergovernmental organisations ([M’barek *et al.*, 2017](#_ENREF_7); [Cui *et al.*, 2018](#_ENREF_1); [Kuiper *et al.*, 2018](#_ENREF_6); [OECD, 2019](#_ENREF_9)).

The model employs the typical tenets of neoclassical economic behaviour to derive the behaviour of agents. Input demands are subject to the minimisation of costs subject to constant returns to scale production technologies, whilst the regional household optimises its utility subject to a budget constraint. Employing linearly homogeneous functional forms and weak separability conditions, multistage budgeting allows for cost minimising optimisation across a series of sub-nests that permits a parsimonious, yet flexible, treatment of production technologies. Additional market clearing and accounting equations enforce the underlying conditions of the model database, namely that supply equal demand in each market, economic profits remains zero and that the value of output, income and expenditure within the macro circular flow remain equal. Furthermore, the flow of transactions of goods and services around the circular flow are supported by price transmission equations with tax/subsidy rates.

To ensure a model solution, the number of equations and endogenous variables (typically prices and quantities) in the model system must be equal – known as the model ‘closure’. Remaining variables (i.e., tax rates, technology changes, endowment changes) are held exogenous. Neoclassical closure is assumed where savings rates are fixed shares of regional income, inter-regional investment is allocated with movements in relative regional rates of return and capital account imbalances (i.e., savings minus investment) are matched by current account movements (i.e., exports minus imports) such that the balance of payments nets to zero. The closure is therefore not only a mathematical requirement for guaranteeing a model solution, but is also serves as a maintained hypothesis regarding the macroeconomic behavioural mechanisms driving the model. Once the modeller successfully replicates the equilibrium conditions inherent within the underlying database (known as 'calibration'), a simulation run consists of imposing a series of targeted exogenous shocks. These shocks could cover projections on macroeconomic variables to characterise a certain time period and/or shocks to specific market variables. In response, the model arrives at a new matrix of equilibrium prices and quantities to satisfy the market clearing and accounting conditions discussed above. In the case of recursive dynamic models which run over multiple time periods (such as MAGNET), the solution from the end of the previous simulation period, forms the starting point of the next period.

**S.1.2 MAGNET Database**

An illustration of the linkages between the existing GTAP activities and the new bio-based sector and commodity splits, is presented in Figure S.1. The non-standard data additions are highlighted in blue and the standard GTAP sectors appear in white. The arrows indicate the directional flows of

Energy crops

Petroleum (blending)

Pellets

Bioelectricity

Electricity:

Coal

Gas

Wind/solar

Nuclear

Hydro/geothermal

Municipal Waste

Electricity distribution

Kerosene

(blending)

Biochemical sugars to polylactic

acid (fermentation)

Biochemical ethanol to polyethylene (fermentation)

Thermochemical plant based conversion

BF1G

Biodiesel

BF1G

Bioethanol

Crude veg oil (from oilseeds)

BF2G BF2G BF2G Lignocellulosic

biokerosene thermochem biochem sugar

Oilcake

Biomass supply

Aviation

Chemicals

DDGS

Processing & Blending

End uses

Intermediate and final demands

Livestock

Crops

Forestry

Residues

**Figure S.1: Overview of bio-based sectors and linkages in MAGNET (Source: Philippidis et al., 2018)**

biomass, whilst the dashed lines show biomass-processing by-products. A detailed discussion of these sectors and the accompanying data sources is available on the web in [Philippidis *et al.* (2018)](#_ENREF_10).

**S.1.3 MAGNET SDG Insights Module**

To achieve a more holistic and coherent approach to policy implementation, the representation of the SDG indicators/metrics within an *ex-ante* global market simulation model provides a unique insight into the synergies or trade-offs in scenarios where several policy instruments and other drivers are operating simultaneously. The MAGNET SDG Insights Module (MAGNET SIM) embeds 60 official and supporting indicators, covering 12 of the 17 SDGs for each of the 140 regions in the database (see Table S.1). The aim is that complex model output is made more accessible through translation into a series of SDG metrics, which is increasingly becoming part of the common language of global impact assessment. In many cases, the generation of *index* and *share* based SDG indicators directly follow from the existing price, quantity and value indicators within the MAGNET database (e.g., food price indices, rural wages, import and export changes, value added shares by industrial classification etc.). Moreover, access to additional non-standard modules and their associated satellite databases, enriches further the suite of available MAGNET SDG metrics to encapsulate *levels* indicators (e.g., employment head, land areas, calorie intake, water volume abstraction, energy production and consumption in million tonnes of oil equivalent).

**Table S.1: Detailed list of SDG indicators in the MAGNET SDG Insights Module**

| **SDG1: End Poverty** |
| --- |
| Per capita utility from private expenditure  Ratio of rural wage to cereal price (food access measure) |
| **SDG2: End hunger** |
| Total factor productivity |
| Average import tariffs on agricultural food products (ad valorem rate) |
| Average export subsidies on agricultural food products (ad valorem rate) |
| Index of import tariffs on agricultural food products |
| Index of export subsidies on agricultural food products |
| Food availability: |
| Domestic food production (primary agriculture) |
| Domestic food production (primary agriculture including fish)  Food imports (primary agriculture including fish)  Food exports (primary agriculture including fish) |
| Calories per capita per day (excluding fish) |
| Calories per capita per day (including fish)  Share of calories from cereals |
| Protein (grams per person per day) from livestock and fish products |
| Protein (grams per person per day) from livestock products |
| Food access: |
| Average household income per capita in thousand USD  Food prices |
| Share of food expenditure in total income  Food consumption and food consumption per capita |
| Food utilisation: share of calories from fruit and vegetables |
| **SDG4: Quality education** |
| Share of skilled labour |
| **SDG6: Water and sanitation** |
| Percentage change in water use over time (in arable sectors) |
| **SDG7: Sustainable energy** |
| Primary energy |
| Renewable energy share in the total energy input consumption |
| Final energy |
| Relative competitiveness of fossil to renewables |
| Energy security: self-sufficiency (value) |
| Energy security: price per energy unit |
| Energy security: value-added contribution of energy  Land devoted to bioenergy  Ratio of value added to net domestic energy use by industry |
| Energy intensity measured in terms of primary energy & GDP  Share of household spending on energy |
| **SDG8: Sustainable economic growth** |
| Annual growth rate of GDP per capita |
| Net trade position  Share of fossil fuels in GDP  Revealed Comparative Advantage  Diversification index  Net trade position  Annual growth rate of real GDP per employed person (Indicator 8.2.1) |
| **SDG9: Resilient infrastructure** |
| Manufacturing value added as a percentage of total value added |
| Manufacturing value added per capita  Manufacturing employment as a percentage of total employment  CO_2_ emissions (tons per unit of value added)  Combustion emissions (tons per unit of value added)  Non-Combustion emissions (tons per unit of value added)  Trade levels  Trade openness |
| **SDG10: Reduce inequality** |
| Labour share of GDP comprising wages  Skills composition  Wage differential skilled:unskilled  Wage differential agriculture:non-agriculture |
| Change in agricultural employment |
| Agricultural employment as a percentage of total employment |
| **SDG12: Sustainable consumption and production patterns** |
| Share of renewables (bio-based and non bio-based) in total energy production  Amount of fossil fuel subsidies per unit of GDP  Amount of fossil fuel subsidies as a share of national expenditure on fossil fuels |
| **SDG13: Climate action** |
| Share of renewables in total energy production  Factor intensities:  Sectoral value added share in total value added  Share of value added in total costs by sector  Number of countries using a biofuel directive  Emissions per unit of GDP/output  Emissions per calorie |
| **SDG14: Conserve and sustainably use marine resources** |
| Fisheries as a value share of GDP |
| **SDG15: Sustainable use of terrestrial ecosystems including forests** |
| Share of non-agricultural land |
| **SDG17: Strengthen the Global Partnership for Sustainable Development** |
| GDP  Total government revenue (by source) as a percentage of GDP  Developing countries’ and least developed countries’ share of global exports  Average tariffs faced by trading partners  Worldwide weighted tariff average |

**S.2 Transition pathways – Drivers.**^[[1]](#footnote-1)^

**S.2.1 Macroeconomic drivers**

For a full discussion of the assumptions driving the three pathways, the reader is encouraged to consult [Keramidas *et al.* (2018)](#_ENREF_5). Tables S.2 and S.3 present the compounded real GDP and population index trends (2011=100) across the five periods for all 13 regions for the non-sustainable transition pathway to 2050.^[[2]](#footnote-2)^ Note, that in Table S.2 calculated from a base year index of 2011 (=100), the final two columns show the deviations in the SUS and SUS+ compound rate of GDP growth to 2050, compared with the NSUS pathway. From the outset, it is assumed that the technological developments in energy generation, energy efficiency gains and the electrification of the global economy inherent within the 2DEG and 1.5DEG transition pathways, will greatly speed up the decoupling of energy usage from economic activity, although this comes at a macroeconomic cost. The assumed evolution of fossil fuel prices in [Keramidas *et al.* (2018)](#_ENREF_5) are taken from the JRC POLES model ([Després *et al.*, 2018](#_ENREF_2)) and are calculated as dollars per barrel of oil equivalent (see Table S.4).

**Table S.2. Real GDP projections in the SUS and SUS+ pathways in 2050 vs the NSUS.**

|  | **GDP**  **($billions)** | **Real GDP growth rates (%)** | | | | | **2050 growth index (2011=100)** | | |
| --- | --- | --- | --- | --- | --- | --- | --- | --- | --- |
|  | **2011** | **2011-2015** | **2015-2020** | **2020-2030** | **2030-2040** | **2040-2050** | | **SUS vs NSUS** | **SUS+ vs NSUS** |
| **USA & Canada** | 17,298 | 9.4 | 10,9 | 18,7 | 21,8 | 17,9 | | -1,2 | -1,1 |
| **Brazil** | 2,475 | 4.2 | 5,0 | 26,5 | 28,1 | 25,0 | | -1,8 | -2,8 |
| **Latin America** | 3,456 | 13.2 | 15,4 | 41,3 | 40,4 | 36,4 | | -2,5 | -4,5 |
| **North Africa** | 605 | 18.6 | 21,1 | 69,9 | 54,8 | 41,0 | | -9,8 | -24,5 |
| **Sub Sah. Africa** | 1,450 | 21.8 | 24,5 | 73,1 | 74,2 | 68,6 | | -14,3 | -35,5 |
| **EU** | 17,643 | 5.7 | 6,8 | 14,3 | 13,9 | 15,6 | | -0,6 | -2,2 |
| **Rest Europe** | 2,652 | 16.1 | 17,8 | 36,5 | 29,9 | 22,0 | | -2,7 | -4,3 |
| **Russia** | 1,866 | 6.1 | 7,0 | 13,4 | 19,3 | 5,1 | | -8,1 | -13,4 |
| **Middle East** | 2,585 | 15.6 | 17,9 | 41,9 | 35,4 | 25,8 | | -5,8 | -15,3 |
| **India** | 1,873 | 34.7 | 35,4 | 100,9 | 65,2 | 50,8 | | -2,6 | -11,7 |
| **China** | 7,306 | 36.1 | 34,7 | 60,0 | 38,9 | 24,9 | | -0,2 | -2,4 |
| **Rest of Asia** | 10,483 | 11.3 | 12,8 | 27,3 | 25,9 | 22,5 | | -1,0 | -2,5 |
| **Oceania** | 1,599 | 12.3 | 14,5 | 31,6 | 31,7 | 27,1 | | -0,2 | -1,4 |

**Source: Keramidas *et al*. (2018), personal communication with GEM-E3 modellers and own calculations.**

**Table S.3. Population projections for the NSUS, SUS and SUS + pathways**

|  | **Population** | **Population (%)** | | | | |
| --- | --- | --- | --- | --- | --- | --- |
|  | **millions** | **2011-2015** | **2015-2020** | **2020-2030** | **2030-2040** | **2040-2050** |
| **USA & Canada** | 346.1 | 2.9 | 11.2 | 6.9 | 5.7 | 4.7 |
| **Brazil** | 196.9 | 3.5 | 5.1 | 5.9 | 3.1 | 1.0 |
| **Latin America** | 404.9 | 4.8 | 15.4 | 8.8 | 6.0 | 3.6 |
| **North Africa** | 166.5 | 7.3 | 21.3 | 11.8 | 8.8 | 6.5 |
| **Sub Sah. Africa** | 878.8 | 11.7 | 24.7 | 24.6 | 20.4 | 16.1 |
| **EU** | 507.8 | 1.0 | 6.9 | 1.6 | 0.8 | -0.0 |
| **Rest Europe** | 243.1 | 3.3 | 17.8 | 4.3 | 2.4 | 1.3 |
| **Russia** | 143.0 | 0.2 | 7.3 | -2.0 | -2.5 | -1.2 |
| **Middle East** | 220.7 | 6.7 | 18.1 | 14.1 | 10.8 | 8.1 |
| **India** | 1,221.2 | 5.0 | 35.2 | 9.6 | 6.5 | 3.9 |
| **China** | 1,344.1 | 1.8 | 34.4 | 0.4 | -3.1 | -5.3 |
| **Rest of Asia** | 1,245.2 | 4.6 | 12.8 | 8.4 | 5.5 | 3.2 |
| **Oceania** | 36.8 | 5.8 | 14.5 | 11.3 | 9.1 | 7.5 |

**Source: Keramidas *et al*. (2018).**

**Table S.4. Fossil fuel prices between 2011 and 2050 ($ per barrel of oil equivalent, 2015 prices)**

| **Fuel type** | **2011** | **2015** | **2020** | **2030** | **2040** | **2050** |
| --- | --- | --- | --- | --- | --- | --- |
| **NSUS pathway** |  |  |  |  |  |  |
| **Coal** | 24.2 | 24.3 | 24.5 | 27.3 | 31.2 | 33.6 |
| **Crude oil** | 86.9 | 88.8 | 91.2 | 89.1 | 96.2 | 106.6 |
| **Gas** | 38.8 | 44.6 | 51.8 | 61.0 | 71.8 | 77.9 |
| **SUS pathway** |  |  |  |  |  |  |
| **Coal** | 24.2 | 24.3 | 24.7 | 28.9 | 33.0 | 37.4 |
| **Crude oil** | 86.9 | 88.8 | 88.8 | 89.1 | 88.4 | 90.2 |
| **Gas** | 38.8 | 44.6 | 51.6 | 62.8 | 71.9 | 75.2 |
| **SUS+ pathway** |  |  |  |  |  |  |
| **Coal** | 24.2 | 24.3 | 24.7 | 29.6 | 55.8 | 65.3 |
| **Crude oil** | 86.9 | 88.8 | 88.0 | 89.1 | 78.6 | 80.7 |
| **Gas** | 38.8 | 44.6 | 51.5 | 63.2 | 71.5 | 74.2 |

**Source: Keramidas *et al*. (2018)**

**S.2.2 Energy market and emissions drivers**

The NSUS pathway assumes that progress is purely driven by market forces and technological progress, with no explicit recognition of additional climate agreements beyond 2017. The more profound energy balance transition pathways in the SUS and SUS+ are motivated by (i) increases in energy efficiency (i.e. decoupling economic growth from energy consumption), (ii) the shifting of energy carriers toward electrification and (iii) the decarbonisation of energy through the adoption of (bio)renewables. In Figure S.2 is presented the global trends for fossil energy consumption and emissions.


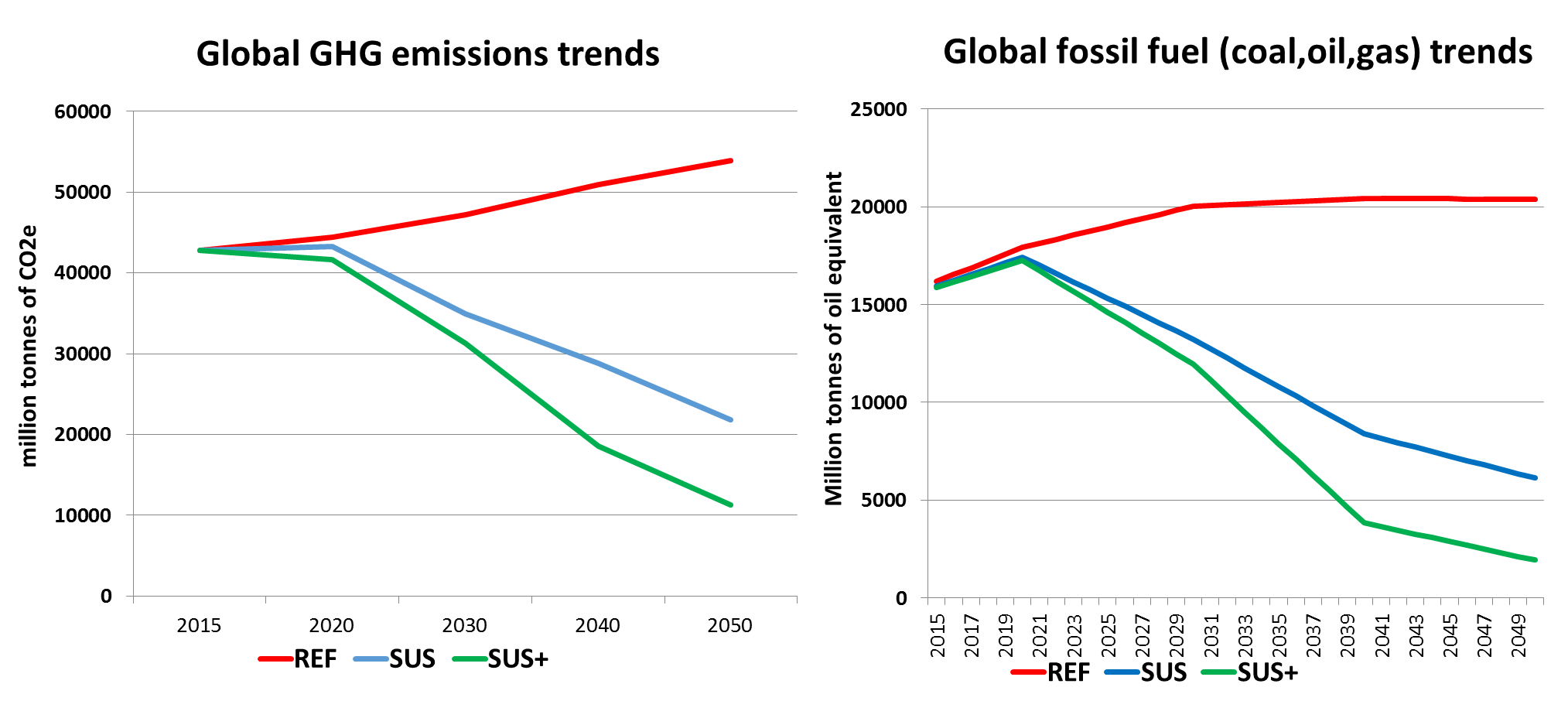


**Figure S.2. Assumed global changes in emissions and energy market trends**

**Source: Own elaboration based on Keramidas *et al*. (2018)**

A cursory examination of the fossil fuel usage trends across the NSUS, SUS and SUS+ transition pathways provides a clear illustration of the decarbonisation in energy usage (particularly in the use of coal). A portion of this is related to the relatively low-cost option of energy efficiency gains through *"appliance efficiency, building insulation, turbomachinery performance and efficiency gains by the electric powertrains versus internal combustion engine (ICE) road vehicles for transport"* ([Keramidas *et al.*, 2018, pp36](#_ENREF_5)), which it is expected, will speed up the decoupling between energy usage and economic growth.

In all three pathways, there are also upward trends in support of the electrification of global final consumption energy needs ([not shown – see Keramidas *et al.*, 2018, pp36](#_ENREF_5)). In particular, with technological changes in the transport sector, it is envisaged that there will be a sharp uptake in electricity usage. In tandem, the portfolio of electricity generation technologies by regions is also expected to radically evolve. The electrification of the global economy is underlined by a sustained and steep decline in fossil based electricity generation technologies in the SUS and SUS+ pathways, a rapid reorientation in the portfolio of electricity generation technologies toward non-biological renewables (i.e., wind, solar, hydroelectric) in all three pathways, and in the SUS and SUS+ narratives (from a low base), a considerably greater reliance on solid biomass for electricity generation.^[[3]](#footnote-3)^

**S.3. Model aggregation**

A detailed regional and commodity aggregation is presented in Table S.5.

**Table S.5. Disaggregation of commodities and regions**

| **Commodity disaggregation** **(59 commodities):**  **Arable and horticulture (9):** paddy rice (pdr); wheat (wht); other grains (grain); oilseeds (oilsd); raw sugar (sug); vegetables, fruits and nuts (hort); other crops (crops); crude vegetable oil (cvol)  **Livestock and meat (7):** cattle and sheep (cattle); pigs and poultry (pigpoul); raw milk (milk); cattle meat (meat); other meat (omeat); dairy (dairy);  **Fertiliser (1):** fertiliser (fert).  **Other food and beverages (2):** sugar processing (sugar); other food and beverages (ofdbv);  **Other ‘traditional’ bio-based (2):** fishing (fish); forestry (frs); wood products (woodpro); paper products (paperpro); textiles & clothing (textcloth).  **Bio-mass supply (12):** energy crops (energy); residue processing (res); pellets (pel); by-product residues from rice (r_pdr); by-product residues from wheat (r_wht); by-product residues from other grains (r_grain); by-product residues from oilseeds (r_oilsd); by-product residues from horticulture (r_hort); by-product residues from other crops (r_crops); by-product residues from forestry (r_frs); Municipal waste (waste).  **Bio-based liquid energy (5):** 1st generation biodiesel (biod); 1^st^ generation bioethanol (biog); 2^nd^ generation thermochemical technology biofuel (ft_fuel); 2^nd^ generation biochemical technology biofuel (eth); bio-kerosene (bkero).  **Bio-based and non-bio-based animal feeds (3):** 1st generation bioethanol by-product distillers dried grains and solubles (ddgs); crude vegetable oil by-product oilcake (oilcake); animal feed (feed).  **Renewable electricity generation (3):** bioelectricity (bioe); hydroelectric (ely_h); solar and wind (ely_w).  **Fossil fuels and other energy markets (10):** crude oil (c_oil); petroleum (petro); gas (gas); gas distribution (gas_dist); coal (coa); coal-fired electricity (ely_c); gas-fired electricity (ely_g); nuclear electricity (ely_n); electricity distribution (ely); kerosene (kero).  **Other sectors (5):** chemicals, rubbers and plastics (crp); other manufacturing (manu); aviation (avi); other transport (trans); services (svcs).  **Regional disaggregation (13 regions):**  USA and Canada (USACAN; Brazil (Bra); Latin America (LatAme); Northern Africa (NoAfrica); Sub Saharan Africa (SSAfrica); European Union (EU); Rest of Europe (REurope); Russia (Rus); Middle East (MidEast); India (Ind); China (Chn); Rest of Asia (RAsia); Oceania (Oce). |
| --- |

**S.4. Modelling transition pathways within the MAGNET model**

With a focus on the implications of different pathways for the SDGs, a full calibration of the MAGNET model to faithfully replicate the detail of the energy balances within Keramidas *et al.* (2018) is beyond the scope of this research. The relative strength of the MAGNET model is its ability to provide indicators from simulation in relation to natural resources, the bio-based economy, food and nutrition. Notwithstanding, the coverage of electricity generation in MAGNET, although perhaps not as detailed as GEM-E3 CGE model used in [Keramidas *et al.* (2018)](#_ENREF_5), is more than adequate to take advantage of the electricity generation pathways. In addition, MAGNET has an emissions module complete with green taxes and a series of accounting and behavioural equations, which link green taxes to the core price transmission equations of the model and also link satellite GTAP data on GHG emissions by sector and region to relevant input usage or output model drivers. Moreover, with its bio-based sector coverage, MAGNET's representation of solid and liquid bioenergy production and usage is state-of-the-art. For each of the three pathways, a three-step calibration approach is followed that is described below.

**S.4.1 First step of calibration**

An initial 'calibration' exercise is performed across the five periods of the study to identify the necessary changes in technology and taste shifters, as well as tax rates to hit specific target variables on quantity usage of different energy types (i.e., fossil, renewables) in intermediate (industrial energy and electricity generation) and final demands, real GDP changes and projected global price developments in the fossil fuel markets.

Thus, in an initial calibration run, real GDP change for each transition pathway is exogenised (swapped with an economy-wide productivity variable) and shocked (see Table S.2 above), whilst population is also exogenously shocked (see Table S.3 above). In MAGNET, the labour force (also exogenous) is assumed to change at the same rate as the population, whilst the long-run observation of fixed capital-output ratios is maintained, such that capital changes exogenously at the same rate as real GDP.

Global fossil fuel (i.e., gas, oil, coal) prices are exogenously targeted (see Table S.4 above) through endogenous adjustments in corresponding global fossil fuel tax variables. Global aggregate data on consumption (million tonnes of oil equivalent) of coal, oil, gas and electricity for agriculture, industry, services^[[4]](#footnote-4)^ and transport is implemented exogenously and is accommodated by endogenous input-output productivity adjustments to target the trends in GECO. The regional share of world electricity generation for each technology calculated from GECO (i.e., hydroelectric, solar-wind composite, bioelectricity, nuclear) is also exogenously controlled through additional input-output efficiency shifters. For the private household, global aggregated trends for coal, oil, gas and electricity energy savings are exogenously implemented by swapping with taste shifters, which redistribute expenditures toward non-energy items.

Global carbon prices for the three transition pathways are taken directly from [Weitzel *et al.* (2019)](#_ENREF_11). They are inserted exogenously into the model through the green tax linkage equations with the core prices in the model. Thus, in the NSUS (non-sustainability), SUS (two degree) and SUS+ (1.5 degree) transition pathways, a global average carbon price in the three transition pathways of between $4-$5 is recorded for 2020, which by 2050, is assumed to reach $47 per tonne, $205 per tonne and $1,205 per tonne (2011 prices), respectively. The resulting endogenous changes in global greenhouse gas combustion and process emissions are recorded in this first simulation exercise.^[[5]](#footnote-5)^

Biofuel mandates on first-generation (1G) and second-generation (2G) in the petroleum (refining) sector and biokerosene mandates in the kerosene (blending) sector are implemented employing exogenous target share rates which are accommodated by endogenous blending subsidies that are met by equal taxes on the users of blended petroleum and kerosene (fiscal neutral). EU mandates follow the plans set out under the EU’s Renewable Energy Directive. Thus, 1G and 2G biofuels blending rates are averaged to 7 and 1.75 percent by 2030^[[6]](#footnote-6)^. In the non EU regions, the mandates follow the assumptions employed in the H2020 ‘SIM4NEXUS’ project for 2020,^[[7]](#footnote-7)^ which are extended to 2030. Beyond 2030, the mandates are allowed to adjust endogenously subject to changing the energy conditions (usage and fossil fuel prices) within each of the three transition pathways. In those cases where the blending rate fell below the 2030 rate, the 2030 blending rate was maintained. In those cases where the blending rate climbed to an implausibly high level, an upper ceiling limit was imposed of twice the 2030 rate. To capture the ‘European Advanced Biofuels Flightpath’ initiative ([IATA, 2015](#_ENREF_4)), biokerosene sold to the kerosene blending sector is assumed to follow the same trend as second generation biofuels in all regions.^[[8]](#footnote-8)^

Finally, as a basis for establishing the long run sustainability conditions underlying biomass availability within each of the transition pathways, a key input is the medium to long run expectations of land productivity change. Unfortunately, this driver is not considered in [Keramidas *et al.* (2018)](#_ENREF_5). As an alternative, this study employs the same assumptions as [Philippidis *et al.* (2018)](#_ENREF_10), which uses land productivity projections by agricultural activity and region taken from shared socio-economic pathway 2 (SSP2) to 2050 from [O’Neill *et al.* (2014)](#_ENREF_8). Whilst it is true that these exogenous SSP2 productivities are unchanged across the three transition pathways, additional endogenous land productivity variable corrections also apply by transition pathway, which result from the differing levels of emissions reductions on temperature change and land productivity (see discussion on ‘damage functions’ in section 3.1 of the main text). These endogenous changes are recorded and exogenised in the final stage of the calibration.

**S.4.2 Second step of calibration**

The final run for each transition pathway implements the same exogenous changes in capital, labour force, population, carbon taxes and land productivities (from SSP2). In addition, calibrated input-output technology and taste 'shifters' from the energy markets, as well as region wide productivity change to meet real GDP forecasts, are now rerun as exogenous shocks. Moreover, the differences between the endogenous reductions in combustion emissions and the required emissions reductions within the pathways of [Keramidas *et al.* (2018)](#_ENREF_5) is met by exogenous reductions in process emissions, which are accompanied by per unit output cost rises to meet the adjustment costs of these transitions.

In the advanced bio-based sectors, a comparison is made between the global change in biofuels usage reported in the model in million tonnes of oil equivalent (mtoe), with the corresponding GECO global projections of biomass usage in transportation across the time periods. The total energy output difference between the two in mtoe is assumed to be met by second generation biofuels in petroleum and biokerosene in aviation^[[9]](#footnote-9)^, which allows us to calibrate total factor productivity (tfp) technology change in these sectors in the second simulation run. It is further assumed that nascent equivalent thermochemical and biochemical technologies also have the same rate of technology change as their bioenergy counterparts.

**S.4.3 Final simulations**

In the final simulation runs, the choice of exogenous and endogenous variables is mostly identical to the second step calibration runs. The key differences are that total factor productivity technology shifters in the advanced generation bio-based sectors calibrated from step two, are now exogenously shocked. Also, ‘damage function’ driven land productivities are also now exogenised and shocked in these simulations.

**S.5. Scenario results**

**S.5.1. Radar plots**

The global radar plots in Figure 2 show the progression towards, or regression away from, nine SDG targets in the three scenarios. The range of each spine is determined by the minimum and maximum of the logged value of the indicator across the NSUS, SUS and SUS+ scenarios in 2030 and 2050, with 2015 located at zero. Values are logged to condense the indicators which have significantly different ranges. Both years are used in the calculation of the range to allow for comparison across the 2030 and 2050 radar plots. The range is then divided into tenths and each pathway result is allocated a place along the scale according to its value within the range. The range shown is +12 to -12 to prevent bunching at the centre. Each indicator is standardised so that an outward movement along the spine indicates progress towards the target and an inward movement along the spine is a regression away from the target.

**S.5.2 Decomposition**

Figures 3, 6, 8 and 9 present decompositions of the difference in the change in the headline variable between 2015-2050 across two scenarios; specifically, income per capita (Figure 4), calories per capita (Figure 6), land area (Figure 9) and water use (Figure 10). The decomposition identifies the part-worth contribution of exogenous drivers to the total net effect using the GEMPACK Subtotals option ([Harrison *et al.*, 2000](#_ENREF_3)).

**S.6. References**

Cui, H.D., Kuiper, M., van Meijl, H., Tabeau, A., 2018. Climate change and global market integration: Implications for global economic activities, agricultural commodities and food security. The state of agricultural commodity markets (SOCO) 2018: Background paper. Rome.

Després, J., Keramidas, K., Schmitz, A., Kitous, A., et al., 2018. Poles-JRC model documentation - updated for 2018. Joint Research Centre 10.2760/814959.

Harrison, W.J., Horridge, J.M., Pearson, K.R., 2000. Decomposing simulation results with respect to exogenous shocks. Computational Economics 15, 227-249. 10.1023/a:1008739609685.

IATA, 2015. Iata sustainable aviation fuel roadmap. [www.iata.org/whatwedo/environment/Documents/safr-1-2015.pdf](file:///\\net1.cec.eu.int\JRC_NEW\JRC.D\JRC.D.4\SCIENTIFIC\BIOECONOMY\20%20Our_publications\20%20Peer_reviewed_articles\2020_JCP_MAGNET-SDGs\www.iata.org\whatwedo\environment\Documents\safr-1-2015.pdf).

Keramidas, K., Tchung-Ming, S., Diaz-Vazquez, A.R., Weitzel, M., et al., 2018. Global energy and climate outlook 2018: Sectoral mitigation options towards a low-emissions economy – global context to the eu strategy for long-term greenhouse gas emissions reduction. Publications Office of the European Union, Luxembourg.

Kuiper, M., Shutes, L., Verma, M., Tabeau, A., et al., 2018. Exploring the impact of alternative population projections on prices, growth and poverty developments. Background paper to the UNCTAD-FAO commodities and development report 2017, commodity markets, economic growth and development.

M’Barek, R., Barreiro-Hurle, J., Boulanger, P., Caivano, A., et al., 2017. Scenar 2030 - pathways for the European agriculture and food sector beyond 2020. EUR 28883 EN. Luxembourg.

O’Neill, B.C., Kriegler, E., Riahi, K., Ebi, K.L., et al., 2014. A new scenario framework for climate change research: The concept of shared socioeconomic pathways. Clim. Change 122, 387-400. 10.1007/s10584-013-0905-2.

OECD, 2019. A global economic evaluation of GHG mitigation policies for agriculture [com/tad/ca/env/epoc(2018)7/final].

Philippidis, G., Bartelings, H., Helming, J., M'Barek, R., et al., 2018. The magnet model framework for assessing policy coherence and sdgs: Application to the bioeconomy. European Commission - Joint Research Center, Luxembourg 10.2760/560977.

Weitzel, M., Vandyck, T., Keramidas, K., Amann, M., et al., 2019. Model-based assessments for long-term climate strategies. Nature Clim. Change 9, 345-347. 10.1038/s41558-019-0453-5.

Woltjer, G.B., Kuiper, M., 2014. The Magnet model: Module description. LEI Wageningen UR, The Hague, The Netherlands.

1. In addition to the shocks described here, additional background trade shocks for the EU are implemented in the 2011 to 2015 (first) period to finalise the accession of Croatia to the European Union. [↑](#footnote-ref-1)
2. With a different regional concordance employed between the study of Keramidas *et al*. (2018) and this study, additional side programs were coded to map the assumed real GDP and population trends to fit the regional aggregation of choice employed within this study. [↑](#footnote-ref-2)
3. In the absence of more detailed data from Keramidas *et al*. (2018), in the MAGNET model transition pathways, it is assumed that electricity generation from pellets and solid municipal waste change at the same rate. [↑](#footnote-ref-3)
4. In the GECO data (Keramidas *et al*. 2018), the term 'Buildings' is used to capture both services and residential usage of energy. [↑](#footnote-ref-4)
5. It should be noted that there is no explicit Carbon Capture and Utilisation (CCU) mechanism modelled in MAGNET, therefore the global emissions reductions are set to match the targets in Keramidas *et al*. (2018) with the contribution of CCS. [↑](#footnote-ref-5)
6. 2G mandates are double counted. [↑](#footnote-ref-6)
7. https://www.sim4nexus.eu/index.php?wert=Home [↑](#footnote-ref-7)
8. The 0.5% assumption by 2030 should be considered as an upper limit. Indeed, given the cost disadvantage of biokerosene next to fossil based kerosene (IATA, 2015), it is noted in that, “…only after 2035 (will) biofuels (bio-kerosene) slowly start penetrating the aviation fuel mix” (IATA, 2015, pp64). [↑](#footnote-ref-8)
9. In the NSUS pathway, the increase in 2G biofuels is negligible to meet the GECO target, whilst in the spirit of the SUS and SUS+ narratives, where sustainable biofuel use 2G technologies, the required increases in 2G biofuels up to 2050 are considerably larger. [↑](#footnote-ref-9)
